# Moderate Wine Consumption Reduces Faecal Water Cytotoxicity in Healthy Volunteers

**DOI:** 10.3390/nu12092716

**Published:** 2020-09-05

**Authors:** Irene Zorraquín-Peña, Dolores González de Llano, Alba Tamargo, M. Victoria Moreno-Arribas, Begoña Bartolomé

**Affiliations:** Institute of Food Science Research (CIAL), CSIC-UAM Nicolás Cabrera, 9. Campus de Cantoblanco, 28049 Madrid, Spain; irene.zorraquin@csic.es (I.Z.-P.); d.g.dellano@csic.es (D.G.d.L.); alba.tamargo@csic.es (A.T.); victoria.moreno@csic.es (M.V.M.-A.)

**Keywords:** faecal water cytotoxicity, wine, HT-29, HCT 116, SCFA, phenolic acids

## Abstract

There are some studies that suggest that moderate consumption of wine, as part of a healthy and balanced diet, has a favourable effect on intestinal health. This study evaluates the effect of moderate wine consumption on faecal water (FW) cytotoxicity as a parameter of gut health. To that end, faecal samples before and after a red wine intervention study (250 mL of wine/day, 4 weeks) in healthy volunteers (*n* = 8) and in a parallel control group (*n* = 3) were collected and assayed for in vitro FW cytotoxicity. Two reference compounds, phenol and *p*-cresol, were used for assessing the cytotoxicity assays using two colon epithelial cell lines (HT-29 and HCT 116) and different assay conditions (FW dilution and incubation time). For the two cell lines and all assay conditions, the means of percentage cell viability were higher (lower cytotoxicity) for samples collected after the red wine intervention than for those collected before, although significant (*p* < 0.05) differences were only found in certain assay conditions for both cell lines. Significant positive correlations between the percentage cell viability and the contents of some faecal metabolites (short-chain fatty acids (SCFA) and phenolic acids (PA)) were found for the more resistant cell line (HCT 116), suggesting that the reduction in FW cytotoxicity observed after moderate red wine consumption was related to the production of microbial-derived metabolites such as SCFA and PA, whose faecal contents have been shown to increase after wine consumption. FW cytotoxicity can be deemed as a holistic biomarker that involves diet, gut microbiota and host.

## 1. Introduction

Wine is considered one of the foods with the highest content of polyphenols, to which the health effects associated with moderate consumption of wine are mainly attributed. Although the greatest accumulation of polyphenols and phenolic metabolites (i.e., microbial-derived metabolites) takes place at the intestinal level, studies on the effects of wine on human digestive function and health are still scarce [1].

The colon lumen harbours metabolites resulting from food fermentation, the inhabitant microbiota and colonic cells. Gut microbiota enable the metabolism of both dietary and endogenous substrates to be used by the host, but microbial-derived metabolites can also modulate the immune system through impacting the physiology and gene expression of host cells [2]. Among dietary macronutrients, carbohydrates, proteins and fat can escape from primary digestion and reach the colon, where they are catalysed into metabolites of a different nature [3,4]. The bioavailability of micronutrients, such as different water-soluble vitamins, is influenced by gut microbiota, as gut bacteria can synthesise them [5]. Additionally, gut microbiota enable extensive metabolism of non-nutrients such as polyphenols, whose metabolites may exert their action at the gut level and at the systemic level after absorption by the host [1]. As an approach to studying the role of colonic metabolites inside the lumen, researchers have been working with what it is called “faecal water” (FW), which is the aqueous phase obtained after faeces ultracentrifugation [6]. The rationale behind this approach is that components of the FW fraction are more likely to exert their effects on the colonic mucosa than substances bound to insoluble food residues or colonic bacteria [7]. Several studies have demonstrated that FW is cytotoxic to mammalian cells in culture, and, based on this, “FW cytotoxicity” has been considered as a parameter of gut health [8,9]. FW has been shown to exert, in cultured cells, cytotoxic, genotoxic and other effects that have implications for colorectal cancer risk [7]. FW contains a range of metabolites derived from dietary sources that might be implicated in the initiation and/or development of colorectal cancer, such as bile acids, fatty acids, N-nitroso compounds and heterocyclic amines, as well as compounds that are potentially beneficial, including short-chain fatty acids (SCFAs) and polyphenols [8]. Additionally, FW cytotoxicity has been associated with microbial populations in specific human nutritional situations, as in the study by Beaumont et al. [10], who related “low cytotoxic” with a relative major abundance of Firmicutes and *Ruminococcaceae* and “high cytotoxic” with a major abundance of Proteobacteria, *Bacteroides* and *Lachnospira* in faecal samples from an intervention study with casein and soy protein. In mice, the increase in FW cytotoxicity observed after dietary supplementation with haem (the iron-porphyrin pigment of red meat) was associated with an increase in Bacteroidetes and a decrease in Firmicutes in colonic contents [11]. On the other hand, several studies have demonstrated that FW cytotoxicity can be modified by diet; in particular, consumption of prebiotics, probiotics, calcium and fibre seems to reduce FW cytotoxicity [8,12]. Intra- and inter-individual variability in FW toxicity (i.e., genotoxicity) has been found to be rather high, and inter-individual variability has not been lowered when subjects have consumed identical diets [13]. However, effective experimental protocols may still lead to detectable modulations of the level of toxic and genotoxic effects, making FW toxicity a suitable biomarker during dietary intervention studies [13].

Therefore, the aim of this work was to assess the changes in the biomarker “FW cytotoxicity” after a wine nutritional intervention in healthy volunteers. To that end, and after an assay assessment using phenol and p-cresol as reference compounds, faecal samples before and after a red wine intervention study (250 mL of wine/day, 4 weeks) in healthy volunteers (n = 8) and in a parallel control group (*n* = 3) were collected and assayed for FW cytotoxicity towards to two colon epithelial cell lines (HT-29 and HCT 116). Then, and in order to establish a possible relationship between FW cytotoxicity and faecal metabolites, Pearson’s correlations between the percentage cell viability and the concentration of two target classes of microbial-derived metabolites (short-chain fatty acids (SCFA) and phenolic acids (PA)) were carried out.

## 2. Materials and Methods

### 2.1. Phenolic Compounds

Phenol and *p*-cresol were obtained from Sigma-Aldrich (St. Louis, MO, USA) and Alfa Aesar (Ward Hill, MA, USA), respectively. Solutions of phenol (50, 25, 10, 5, 0.05, 0.025 and 0.005 mM) and p-cresol (7.40, 3.70, 1.85, 0.92, 0.46 and 0.09 mM) compounds were prepared in cell culture medium.

### 2.2. Red Wine

A young red wine (Pinot noir, vintage 2010) was selected for the intervention study. The selection was based on its high phenolic compounds and good organoleptic properties. The ethanol content in the wine was 12%. The total phenolic content reached 1758 mg of gallic acid equivalent/L, comprising total anthocyanins (447 mg of malvidin-3-O-glucoside/L) and total catechins (1612 mg of (+)-catechin/L). The concentration of individual phenolic compounds in this wine is reported elsewhere [14].

### 2.3. Wine Intervention Study

**Figure 1:**
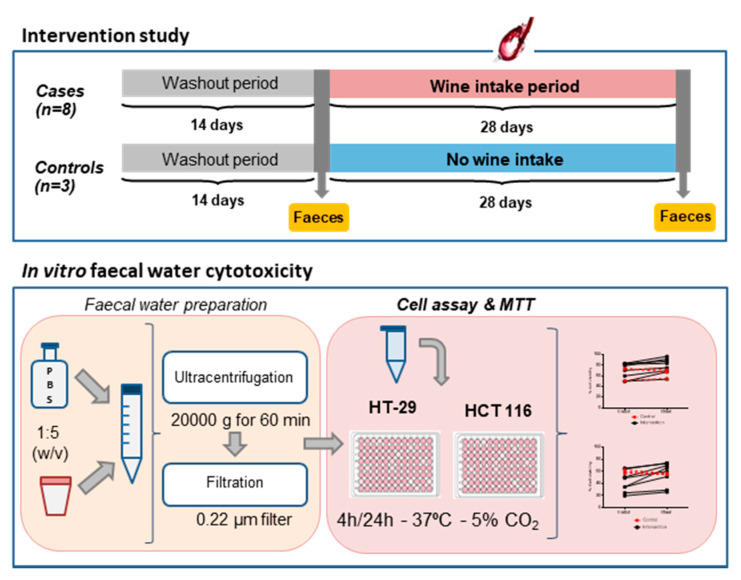
Flow chart of the experimental approach followed in this study.

A six-week randomised controlled trial was carried out [13], according to the rules and approval of the Bioethics Committee of the “Ramón y Cajal” Hospital (Madrid, Spain): AGL2009-13361-C02-00. The participants had not suffered from any disease or intestinal disorder, had not received antibiotics or any other medical treatment and had not consumed any probiotic and/or prebiotic supplements for at least 6 months before the study, and this remained the case during it. All participants were informed about the study and signed an informed consent form. The study was divided into two consecutive periods: (1) an initial washout period of 2 weeks during which the volunteers did not consume wine or any other alcoholic beverage and followed a diet low in polyphenols; and (2) a period of 4 weeks during which case volunteers (n = 8) consumed a daily intake of red wine (250 mL/two doses), while control volunteers (n = 3) kept to the low-polyphenol diet without wine consumption. The case group was formed by four women and four men (age range 20–65 years) and the control group by two women and one man (age range 30–50 years). Daily intake of red wine (250 mL) was established in previous studies [14]. Faecal samples were collected at two points: at the end of the washout period (t_initial_) and at the end of the intervention period (t_final_) (Figure 1). Faeces were immediately frozen and stored at −80 °C until being analysed.

### 2.4. Faecal Water Preparation

Faecal water (FW) was prepared as indicated in Figure 1. Faeces were defrosted and diluted 1:5 (*w*/*v*) in Dulbecco’s phosphate-buffered solution (DPBS) (Sigma-Aldrich, St. Louis, MO, USA). Then, they were ultracentrifuged at 20,000 g for 60 min (Beckman Coulter, Brea, CA, USA) and the supernatants were filtered through 0.22 µm PVDF filters (Symta, Madrid, Spain). Three dilutions—1:2, 1:5 and 1:10—of the faecal water were made with serum-free cell culture medium.

### 2.5. Cell Cytotoxicity Assay

Two human colon adenocarcinoma cell lines, HT-29 (ATCC^®^ HTB-38™) and HCT 116 (ATCC^®^ CCL-247™), were used to evaluate cell cytotoxicity. HT-29 cells were cultured in McCoy’s 5A with L-glutamine (Lonza, Basel, Switzerland) supplemented with 1% (*v/v*) penicillin/streptomycin solution (Sigma-Aldrich, St. Louis, MO, USA) and 10% (*v/v*) foetal bovine serum (FBS; Biowest Europe, Nuaillé, France). HCT 116 cells were cultured in Dulbecco’s modified Eagle’s medium (DMEN), high glucose (4.5 g/L) (Lonza, Basel, Switzerland) supplemented with 1% (*v/v*) penicillin/streptomycin solution and 10% (*v/v*) FBS. Cells were grown in 75 cm^2^ flasks (Corning Flask, Corning, NY, USA) at 37 °C and in a 5% CO_2_ atmosphere and the media were renewed every 3 days.

Cytotoxicity against HT-29 and HCT 116 cells was measured using the colorimetric MTT assay, which is based on the reduction of 3-(4,5-dimethylthiazol-2-yl)-2,5-diphenyltetrazolium bromide (MTT) to formazan, an insoluble intracellular blue product, by cellular dehydrogenases. Cells were seeded at a density of 3.6 × 105 cells/mL on well plates, and washed with warm DPBS. Then, they were incubated (100 µL/well) with diluted FW, phenolic compound solutions or serum-free medium (used as control) for 4 or 24 h. After that, supernatants were removed from the wells, and MTT (Sigma-Aldrich, St. Louis, MO, USA) solution (0.5 mg/mL) was added (100 µL/well). The plates were immediately incubated for 3 h in darkness. After this time, MTT reagent was aspirated and formazan crystals were diluted in 100 µL of dimethyl sulfoxide (DMSO; Sigma-Aldrich, St. Louis, MO, USA). Absorbance at 570 nm was measured on a Multiskan plate reader (Thermo Scientific, Newington, NH, USA). Cell viability (%) was calculated as Abssample/Abscontrol × 100. Assays were performed in triplicate and three independent experiments were carried out.

IC_50_ (half maximal inhibitory concentration) values for phenol and *p*-cresol were estimated using a sigmoidal dose-response curve with variable slope, utilising the software Prism 6 (GraphPad Software Inc., San Diego, CA, USA).

### 2.6. Phenolic Acids Analysis 

Phenolic metabolites were determined by ultra-performance liquid chromatography-electrospray ionization-tandem mass spectrometry (UPLC-ESI-MS/MS) following a previously reported method [15]. The liquid chromatographic equipment used was a Waters Acquity UPLC (Milford, MA, USA) system with a binary pump, an autosampler thermostatted at 10 °C and a heated column compartment (40 °C). Details about chromatographic and detection conditions as well as data processing are extensively reported in [15]. Analyses were carried out in duplicate.

### 2.7. Short-Chain Fatty Acids Analysis

Short-chain fatty acids (SCFA) were determined by Solid Phase Micro-extraction-Gas Cromatography-mass spectrometry (SPME-GCMS) following the method developed previously as reported by Cueva et al. [16]. The extraction procedure was automatically performed by using a CombiPAL system (CTC Analytics AG, Zwingen, Switzerland) and desorption was performed using the injector of the GC-MS system (Agilent 7890A, Agilent 5975C MS;Agilent, Santa Clara, CA, USA). Details about chromatographic and detection conditions as well as data processing are extensively reported in Cueva et al. [16]. Analyses were carried out in duplicate.

### 2.8. Statistical Analysis

The statistical analysis was performed using IBM SPSS Statistics v26 for Windows (IBM Corp., Armonk, NY, USA). Paired-sample T-tests for assessing significant differences in cell viability (%) between faecal samples before (t_initial_) and after (t_final_) red wine intervention were performed. Before–after graphs were made using GraphPad Software v6.0 (GraphPad, San Diego, CA, USA). Pearson’s correlation coefficients between the percentage of cell viability and faecal content in fatty acids and phenolic metabolites were calculated. A value of *p* = 0.05 was fixed for the level of significance of the statistical analysis.

## 3. Results and Discussion

### 3.1. Assessment of the FW Cytotoxicity Assays

Cytotoxicity assays using two colon epithelial cell lines (HT-29 and HCT 116) and two incubation times (4 and 24 h) were carried out in an effort to reproduce the physiological conditions as faithfully as possible. Two compounds, phenol and *p*-cresol, which arise from bacterial action on dietary aromatic amino acids and are normally present in faeces [17], were used as reference compounds for the assessment of the cytotoxicity assays. Both compounds, together with other microbial-derived metabolites, may be involved in the modulation of chronic bowel inflammation, tissue permeability and colitis severity in the gut [18]. Table 1 reports the half-maximal inhibitory concentrations (IC_50_) of phenol and *p*-cresol against both HT-29 and HCT 116 cell lines. Greater toxicity (lower IC_50_ values) was observed for *p*-cresol than for phenol, and HT-29 cells were found to be more sensitive to both compounds than HCT 116 cells (Table 1). As expected, assay sensitivity increased (lower IC_50_ values) from 4 to 24 h incubations for both compounds and cell lines, but IC_50_ values still remained in the mM range (Table 1). Our results were in accordance with those of Andriamihaja et al. [19], who confirmed the cytotoxicity effects for *p*-cresol against the HT-29 Glc–/þ cell line after 24 h incubation at a concentration of 0.8 mM. Similarly, it was observed that *p*-cresol exerted a cytotoxic effect from 3 mM towards HT-29 and Caco 2 cells after 24 h of incubation [20]. Among other phenols, Pedersen et al. [21] found that, in particular, phenol became cytotoxic against HT-29 cells at a concentration of 1.25 mM, which was in line with our results. We have found no references relative to the toxic effects of phenol or *p*-cresol against the HCT 116 cell line.

In summary, these results were consistent with previous data reported in the bibliography that showed a direct toxic effect of phenol and *p*-cresol on human colonic epithelial cells in vitro. Additionally, these first results with reference compounds confirmed the suitability of the proposed assays for the assessment of FW toxicity.

### 3.2. Evaluation of FW Cytotoxicity before and after Moderate Red Wine Consumption 

**Figure 2:**
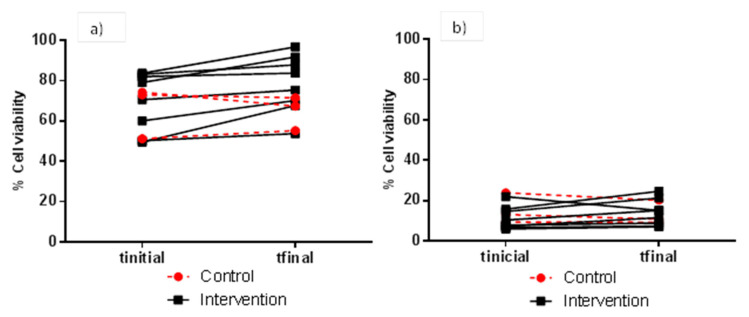
Individual evolution of viability (% with respect to the control) of HT-29 cells after exposure to FW from the control and intervention groups. Cytotoxicity assay conditions: 1:2 (*v/v*) for FW dilution, and 4 h (**a**) and 24 h (**b**) for incubation time.

FW cytotoxicity to HT-29 and HCT 116 cells (as means of percentage cell viability) of faecal samples collected before and after red wine intervention for the control (*n* = 3) and case (*n* = 8) groups is depicted in Table 2 and Table 3, respectively. In general, and for the two cell lines and all assay conditions (FW dilution and incubation time), means of percentage cell viability were higher for FW samples collected after (t_final_) than before (t_initial_) red wine intervention for the case group, although significant differences (*p* < 0.05) were only found in certain assays conditions. As regards the HT-29 cell line, the assay conditions of 1:2 (*v/v*) for FW dilution and 4 h for incubation time showed the highest difference in percentage cell viability (mean values) when comparing samples before (69.8%) and after (78.4%) the red wine intervention (Table 2). Figure 2 details the individual evolution of the percentage cell viability of HT-29 cells measured under these latter conditions, ranging from 51.3 to 74.4% for the intervention group, and 49.5 to 96.8% for the control group. When HT-29 cells were exposed to longer incubation times (24 h), the range of percentage cell viability was much lower and no significant differences (*p* > 0.05) between samples collected before and after the red wine intervention were noted (Table 2).

**Figure 3:**
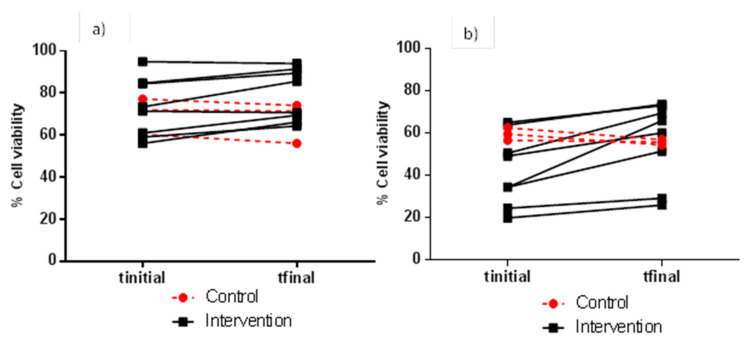
Individual evolution of viability (% with respect to the control) of HCT 116 cells after exposure to FW from the control and intervention groups. Cytotoxicity assay conditions: 1:5 (*v/v*) for FW dilution, and 4 h (**a**) and 24 h (**b**) for incubation time.

As for the more resistant cell line (HCT 116), it was the combination of an FW dilution of 1:5 (*v/v*) and an incubation time of 24 h that led to the highest difference in percentage cell viability (mean values) when comparing samples before (42.7%) and after (56.1%) the red wine intervention (Table 3). Figure 3 details the individual evolution of the percentage cell viability of HCT 116 cells measured under these optimum conditions, ranging from 19.9 to 73.7% for the intervention group, and 54.5 to 62.6% for the control group. At this point, the importance of testing different assay conditions (i.e., FW dilution and incubation time) to ensure the maximum sensitivity of a certain cell line assay in evaluating FW cytotoxicity, and consequently, the maximum feasibility of the assay as a marker of bowel health after nutritional interventions should be noted. Calculating indexes such as EC_25_ (effective concentration at 25% reduction of cell viability) for FW [22] could be useful in this matter.

Our results from both HT-29 and HCT 116 cell models showed a reduction in FW cytotoxicity for the volunteers that consumed red wine (250 mL/day) over a period of 4 weeks, which suggested a protective effect derived from moderate wine consumption in the intestinal lumen. To the best of our knowledge, this is the first study to report data on FW cytotoxicity after the intake of wine or wine-related products in humans. We have only found one related reference, Bastide et al. [23], who reported data from some trials in rats fed with meat supplemented with different plant extracts, including those from white grapes and red wine. They measured FW cytotoxicity to a cancerous mouse colonic epithelial cell line (CMT93), but no changes were found for the diets supplemented with grape or wine extracts, in spite of the fact that other FW biomarkers such as haem and TBARS (thiobarbituric acid reactive substances) were favourably modified [23].

Several studies have investigated how diet could modulate FW cytotoxicity, as a biomarker of bowel health. Among others, prebiotics have gained special attention. Adebola et al. [24] found a 40% increase in HT-29 cell viability when cells were incubated with FW in the presence of prebiotics (inulin and lactulose); these compounds also decreased FW genotoxicity and exerted a protective effect against bile acids. Windey et al. [25] conducted a double-blind randomised controlled trial with humans in which volunteers consumed a wheat bran extract for 2 weeks; FW cytotoxicity, but not genotoxicity, towards HT-29 cells was significantly lower after the intervention. Similarly, Wu et al. [26] conducted an intervention trial to evaluate the effects of Konjac glucomannan (KGM) on precancerous markers of colon cancer. They observed that the per cent viability of Caco-2 cells co-incubated with FW for 1 and 3 h was higher for those samples collected after than before the KGM intervention period (4 weeks) [26]. On the other hand, in another study, no significant differences in FW toxicity towards human enterocytes were observed after consumption of fructo-oligosaccharides (FOS), although it significantly altered bacterial fermentation (higher percentage of acetate, lower percentage of butyrate) [27].

The use of probiotics for reverting toxic effects of microbial-derived and host metabolites has also been tested by evaluating FW cytotoxicity. Klewicka et al. [28] found that, in rats, the intake of beet juice fermented with two *Lactobacillus* strains led to significantly lower FW cytotoxicity, measured in Caco-2 cells. In another study, the FW cytotoxicity towards Caco-2 cells decreased after feeding chickens with a probiotic preparation [29]. In contrast, no significant changes in FW toxicity towards HT-29 cells were observed after a human nutritional intervention with probiotic yogurt [30]. In the case of FW genotoxicity, there are studies that show the protective effect of probiotics such as Bifidobacterium and Lactobacillus strains, among others [31]. In relation to other FW biomarkers, recently, Oliva et al. [32] demonstrated that a 7-day probiotic supplementation also increased the faecal activity against multi-drug-resistant microorganisms in a pilot study that included healthy individuals (n = 6).

In terms of the whole diet, Federici et al. [12], studied the composition of viable faecal bacteria and gut toxicology biomarkers of healthy volunteers, who followed omnivorous, lacto-ovo-vegetarian or vegan diets. They found that the lacto-ovo-vegetarian diet, a less restrictive dietary pattern than the vegan one, was particularly effective in lowering the levels of both FW genotoxicity and cytotoxicity [12]. In a position paper about the use of high-protein diets for weight management [33], the authors concluded that there was no reported increase in FW cytotoxicity or genotoxicity from volunteers consuming high-protein diets in comparison to volunteers consuming normoproteic diets in the short and medium terms. However, studies of whole diets are complex since there are numerous compounds and metabolites derived from digestion. Despite the difficulties of analysis, it seems that compounds derived from plant-based foods can have a positive impact on cytotoxicity and cellular genotoxicity, thereby functioning as modulators of possible diseases [34].

In summary, our results indicate that moderate red wine consumption reduces FW cytotoxicity in vitro, as has been seen for other foods (prebiotics, probiotics, etc.) or whole diets. In other words, moderate red wine consumption modified the luminal metabolite content in such a way that it resulted in lower FW cytotoxicity, with this effect being extremely individual-variable. On the other hand, the fact of selecting healthy volunteers that had not suffered from any disease or intestinal disorders was relevant in the sense that it confirmed the preventive action of moderate wine consumption in the development of certain intestinal diseases.

### 3.3. Correlation Between Cytotoxicity and Some Targeted Metabolites in FW

Previous studies have shown great inter-individual variability in the faecal metabolic profile (i.e., metabolome) after moderate red wine consumption [14,35], which seems to be further derived from differences in intestinal microbiota functionality and/or composition among subjects [36]. In order to establish a possible relationship between FW cytotoxicity and faecal metabolites, Pearson’s correlations between the percentage cell viability and the concentration of two target classes of faecal microbial-derived metabolites (short-chain fatty acids (SCFA) and phenolic acids (PA)) were carried out. For percentage cell viability, data corresponding to assay conditions (i.e., FW dilution) that led to maximum differences between samples collected before and after the red wine intervention were considered (1:2 (*v/v*) for FW dilution in the HT-29 assay and 1:5 (*v/v*) in the HCT 116 assay; Table 2 and Table 3, respectively). Target SCFA included propionic, butyric, isobutyric and isovaleric acids [16]. Target PA included benzoic, phenylacetic, phenylpropionic and cinnamic acids [14]. For both classes of compounds, significant (*p* < 0.05) positive correlations were found for the more resistant cell line (HCT 116) (Table 4).

**Figure 4:**
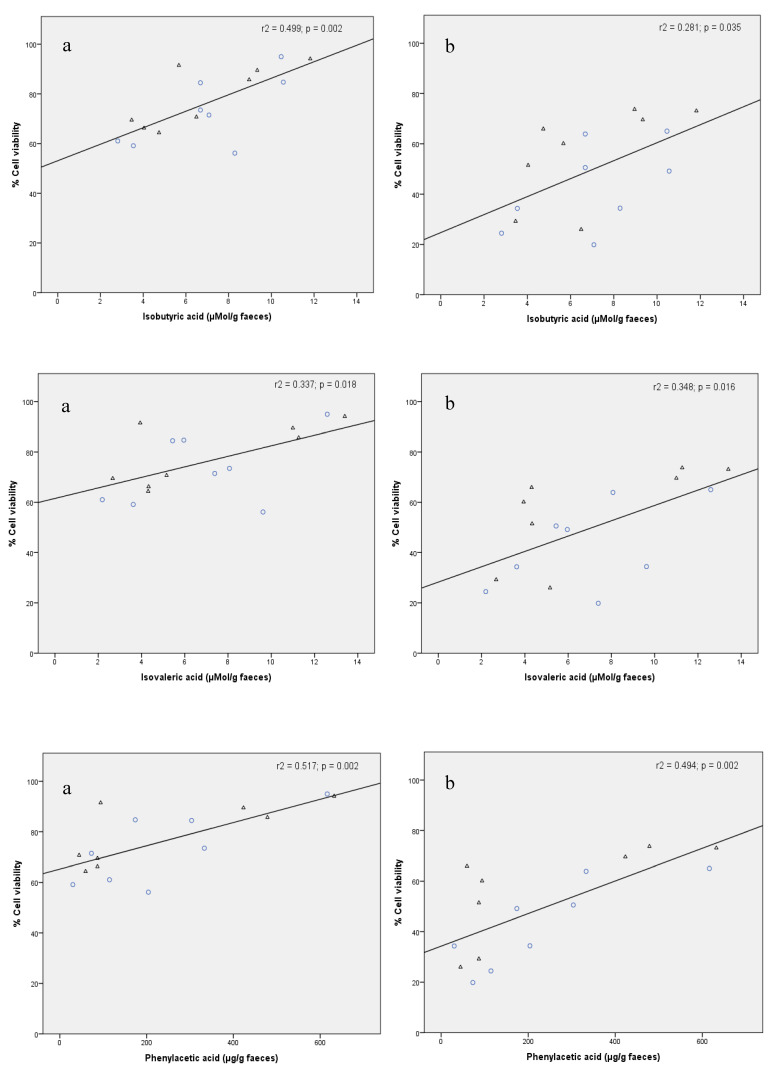
Correlation plots between FW cytotoxicity (percentage viability of HCT 116 cells) and concentration of isobutyric acid (µmol/g faeces), isovaleric acids (µmol/g faeces) and phenylacetic acid (µg/g faeces) in faecal samples collected from volunteers before (O O) and after (△) the red wine intervention. Cytotoxicity assay conditions: 1:5 (*v/v*) for FW dilution, and 4 (**a**) and 24 h (**b**) for incubation time.

Figure 4 illustrates the correlation plots between FW cytotoxicity (percentage cell viability) to HCT 116 cells and the concentration of isobutyric, isovaleric and phenylacetic acids in faeces from the intervention group before and after the red wine intervention (n = 16). Therefore, lower FW toxicity was accompanied by higher levels of certain SCFA and phenolic metabolites. This could be due to the favourable effect of these polyphenol-derived metabolites on cell growth, as they function as an energy source for epithelial cells [37]. Thus, the degradation of dietary fibre and the subsequent fermentation of monosaccharides to SCFA is one of the most widely discussed mechanisms of how gut bacteria impact host physiology [3]. A low concentration of SCFA (specifically acetic, butyric, valeric, isobutyric and isovaleric acids) usually indicates some type of microbial dysbiosis associated with some pathologies such as colorectal cancer and coeliac disease [38]. Thus, the production of fatty acids due to the consumption of certain foods could contribute to the maintenance of intestinal homeostasis.

In summary, these results suggested that the reduction in FW cytotoxicity observed after moderate red wine consumption seems to be related to the production of microbial-derived metabolites such as SCFA and PA, whose faecal contents have been shown to be increased after red wine consumption [14]. In any case, further studies involving a higher number of volunteers and correlations with other metabolomic and metagenomic indexes, such as bile acids composition, should be carried out to expand these findings.

## 4. Conclusions

This paper reports value data of FW cytotoxicity for reference compounds (phenol and *p*-cresol) that can be useful in the development and validation of new protocols. From a methodology point of view, the use of the HTC 116 cell line is also relevant, as these results may be complementary to other cell lines such as HT-29, widely used in cytotoxic and genotoxic tests. The results from a first intervention study (n = 11) concerning this matter indicated that moderate red wine consumption seemed to reduce FW cytotoxicity, with this effect being extremely individual-dependent. FW cytotoxicity was found to be inversely correlated with faecal concentrations of some microbial-derived metabolites such as SCFA and PA, compounds that have been found to be extensively generated after red wine consumption. Therefore, FW cytotoxicity could be seen as a holistic biomarker that involves diet, gut microbiota and host, and can be useful in evaluating nutritional interventions that promote intestinal health maintenance or improvement. Moderate red wine consumption has long been associated with beneficial effects in cardiovascular disease, but the findings of this paper should encourage further studies concerning the effects of moderate red wine consumption on intestinal health, also involving gut microbiota-related diseases such as cognitive and neurodegenerative pathologies.

## Figures and Tables

**Table 1:** Toxicity (IC_50_ value, mM) of phenol and *p*-cresol to HT-29 and HCT 116 cells.

	Phenol		*p*-Cresol	
Time	HT-29	HCT 116	HT-29	HCT 116
4 h	15.8	23.84	5.40	5.64
24 h	6.16	11.41	2.13	4.16

**Table 2:** Viability (% with respect to the control) of HT-29 cells after exposure to faecal water (FW) from the control and intervention groups before (t_initial_) and after (t_final_) wine consumption. Data represent the mean and standard deviation (SD) of three independent experiments, each analysed in triplicate.

		Control Group (*n* = 3)			Intervention Group (*n* = 8)		
		t_initial_	t_final_	*p* Value *	t_initial_	t_final_	*p* Value *
Time	Dilution	Mean ± SD	Mean ± SD		Mean ± SD	Mean ± SD	
4 h	1:1	13.9 ± 2.57	16.3 ± 7.39	0.556	21.0 ± 22.0	23.3 ± 19.8	0.081
	1:2	66.2 ± 12.9	64.7 ± 8.46	0.272	69.8 ± 14.6	78.4 ± 14.3	0.004 *
	1:5	82.7 ± 8.17	81.2 ± 4.09	0.808	88.7 ± 6.31	91.2 ± 6.03	0.047 *
	1:10	91.4 ± 2.55	89.7 ± 4.91	0.568	93.1 ± 3.96	96.7 ± 2.83	0.020 *
24 h	1:1	6.29 ± 1.88	11.3 ± 6.37	0.193	5.51 ± 0.63	6.39 ± 0.80	0.005 *
	1:2	15.5 ± 7.54	15.2 ± 5.5	0.194	11.4 ± 5.66	14.0 ± 6.47	0.169
	1:5	36.2 ± 16.5	41.8 ± 24.7	0.358	26.4 ± 22.8	32.4 ± 21.1	0.114
	1:10	60.7 ± 21.7	64.1 ± 28.4	0.530	48.2 ± 23.9	54.0 ± 21.6	0.072

* Significant differences (*p* < 0.05).

**Table 3:** Viability (% with respect to the control) of HCT 116 cells after exposure to FW from the control and intervention groups before (t_initial_) and after (t_final_) wine consumption. Data represent the mean and standard deviation (SD) of three independent experiments, each analysed in triplicate.

		Control Group (*n* = 3)			Intervention Group (*n* = 8)		
		t_initial_	t_final_	*p* Value *	t_initial_	t_final_	*p* Value *
Time	Dilution	Mean ± SD	Mean ± SD		Mean ± SD	Mean ± SD	
4 h	1:1	26.9 ± 3.62	27.1 ± 7.1	0.962	27.2 ± 17.1	27.9 ± 16.8	0.806
	1:2	62.5 ± 7.52	58.6 ± 5.1	0.343	59.0 ± 19.4	62.4 ± 14.6	0.514
	1:5	69.9 ± 8.62	67.3 ± 9.7	0.143	73.2 ± 14.0	78.9 ± 12.4	0.011 *
	1:10	76.6 ± 3.04	73.9 ± 8.1	0.563	81.0 ± 11.8	84.0 ± 13.0	0.079
24 h	1:1	11.9 ± 5.00	12.6 ± 5.6	0.234	8.92 ± 2.51	10.0 ± 2.73	0.088
	1:2	35.1 ± 17.0	33.9 ± 14.7	0.654	23.9 ± 13.5	34.1 ± 15.2	0.000 *
	1:5	59.6 ± 2.98	55.8 ± 1.3	0.126	42.7 ± 17.1	56.1 ± 19.1	0.004 *
	1:10	71.4 ± 4.765	66.1 ± 3.7	0.148	66.9 ± 18.7	79.0 ± 15.2	0.004 *

* Significant differences (*p* < 0.05).

**Table 4:** Pearson’s correlations between FW cytotoxicity (percentage viability of HT-29 and HCT 116 cells) and concentration of short chain fatty acids (SCFA) (µmol/g faeces) and phenolic acids (PA) (µg/g faeces) in faecal samples (n=16) collected from the intervention group volunteers. Cytotoxicity assay conditions: 1:2 (*v/v*) for FW dilution in HT-29 assay and 1:5 (*v/v*) in HCT 116 assay, and 4 and 24 h for incubation time.

		HT-29 (1:2)		HCT 116 (1:5)	
	Time (h)	R^2^	*p* Value *	R^2^	*p* Value *
*SCFA*					
Acetic acid	4	0.059	0.366	0.121	0.186
	24	0.022	0.585	0.005	0.791
Propionic acid	4	0.020	0.597	0.279	0.035 *
	24	0.006	0.781	0.049	0.408
Butyric acid	4	0.010	0.711	0.163	0.121
	24	0.005	0.788	0.001	0.910
Isobutyric acid	4	0.026	0.511	0.499	0.002 *
	24	0.069	0.327	0.281	0.035 *
Isovaleric acid	4	0.016	0.643	0.337	0.018 *
	24	0.092	0.255	0.348	0.016 *
*Phenolic acids*					
Phenylacetic acid	4	0.100	0.234	0.517	0.002 *
	24	0.143	0.149	0.494	0.002 *
Phenylpropionic acid	4	0.060	0.246	0.357	0.015 *
	24	0.059	0.244	0.048	0.414

* Significant differences (*p* < 0.05).
